# Machine learning derived segmentation of phase velocity encoded cardiovascular magnetic resonance for fully automated aortic flow quantification

**DOI:** 10.1186/s12968-018-0509-0

**Published:** 2019-01-07

**Authors:** Alex Bratt, Jiwon Kim, Meridith Pollie, Ashley N. Beecy, Nathan H. Tehrani, Noel Codella, Rocio Perez-Johnston, Maria Chiara Palumbo, Javid Alakbarli, Wayne Colizza, Ian R. Drexler, Clerio F. Azevedo, Raymond J. Kim, Richard B. Devereux, Jonathan W. Weinsaft

**Affiliations:** 1 Department of Radiology, Weill Cornell Medicine, 525 E 68th St, New York, NY 10065 USA; 2 Greenberg Division of Cardiology, Department of Medicine, Weill Cornell Medicine, 525 E 68th St, New York, NY 10065 USA; 3 IBM TJ Watson Research Center, 1101 Kitchawan Rd, Yorktown Heights, NY 10598 USA; 4 Memorial Sloan Kettering Cancer Center, 1275 York Ave, New York, NY 10065 USA; 5 Duke Cardiovascular Magnetic Resonance Center, 10 Duke Medicine Circle, Durham, NC 27710 USA; 6 Weill Cornell Medical College, 525 East 68th Street, New York, NY 10021 USA

**Keywords:** Cardiovascular magnetic resonance, Machine learning, Deep learning, Phase contrast, Aorta

## Abstract

**Background:**

Phase contrast (PC) cardiovascular magnetic resonance (CMR) is widely employed for flow quantification, but analysis typically requires time consuming manual segmentation which can require human correction. Advances in machine learning have markedly improved automated processing, but have yet to be applied to PC-CMR. This study tested a novel machine learning model for fully automated analysis of PC-CMR aortic flow.

**Methods:**

A machine learning model was designed to track aortic valve borders based on neural network approaches. The model was trained in a derivation cohort encompassing 150 patients who underwent clinical PC-CMR then compared to manual and commercially-available automated segmentation in a prospective validation cohort. Further validation testing was performed in an external cohort acquired from a different site/CMR vendor.

**Results:**

Among 190 coronary artery disease patients prospectively undergoing CMR on commercial scanners (84% 1.5T, 16% 3T), machine learning segmentation was uniformly successful, requiring no human intervention: Segmentation time was < 0.01 min/case (1.2 min for entire dataset); manual segmentation required 3.96 ± 0.36 min/case (12.5 h for entire dataset). Correlations between machine learning and manual segmentation-derived flow approached unity (*r* = 0.99, *p* < 0.001). Machine learning yielded smaller absolute differences with manual segmentation than did commercial automation (1.85 ± 1.80 vs. 3.33 ± 3.18 mL, *p* < 0.01): Nearly all (98%) of cases differed by ≤5 mL between machine learning and manual methods. Among patients without advanced mitral regurgitation, machine learning correlated well (*r* = 0.63, *p* < 0.001) and yielded small differences with cine-CMR stroke volume (∆ 1.3 ± 17.7 mL, *p* = 0.36). Among advanced mitral regurgitation patients, machine learning yielded lower stroke volume than did volumetric cine-CMR (∆ 12.6 ± 20.9 mL, *p* = 0.005), further supporting validity of this method. Among the external validation cohort (*n* = 80) acquired using a different CMR vendor, the algorithm yielded equivalently small differences (∆ 1.39 ± 1.77 mL, *p* = 0.4) and high correlations (*r* = 0.99, *p* < 0.001) with manual segmentation, including similar results in 20 patients with bicuspid or stenotic aortic valve pathology (∆ 1.71 ± 2.25 mL, *p* = 0.25).

**Conclusion:**

Fully automated machine learning PC-CMR segmentation performs robustly for aortic flow quantification - yielding rapid segmentation, small differences with manual segmentation, and identification of differential forward/left ventricular volumetric stroke volume in context of concomitant mitral regurgitation. Findings support use of machine learning for analysis of large scale CMR datasets.

**Electronic supplementary material:**

The online version of this article (10.1186/s12968-018-0509-0) contains supplementary material, which is available to authorized users.

## Background

Cardiovascular magnetic resonance (CMR) is increasingly employed for assessment of patients with known or suspected valvular heart disease. Phase contrast (PC) imaging is central for this application, as it enables quantification of valvular flow and velocity. When acquired through the aortic annulus, PC-CMR also provides an index of left ventricular (LV) stroke volume – enabling LV systolic performance to be measured independent of volumetric analysis. PC-CMR has been well validated in relation to invasive cardiac performance indices, and shown to provide improved predictive value vs. other non-invasive methods such as echocardiography [1–5]. Given widespread utility of PC-CMR, accurate and widely applicable analytic methods for this pulse sequence are of substantial importance.

In routine clinical practice, PC-CMR analysis requires manual segmentation of multiple images (~ 20–30) acquired throughout the cardiac cycle. Given that high temporal resolution is critical for accurate flow quantification, PC-CMR typically entails acquisition of more cardiac frames than do other gated CMR pulse sequences (e.g. cine). Additionally, PC-CMR is often acquired in multiple 2D orientations, adding to cumulative number of images needed for segmentation. Automated PC-CMR segmentation methods are commercially available, but border tracking can be suboptimal – requiring time-consuming operator adjustments that compromise practicality of large-scale studies.

New advances in machine learning have improved capabilities for automated image processing. Convolutional neural networks (CNN) have been a key area of focus, and have been shown to yield human or superhuman performance in a variety of medical classification tasks, including detection of diabetic retinopathy [6] and classification of pulmonary fibrosis [7]. CNN-based segmentation (i.e. pixel-wise classification) models have been applied to CMR, with focus on cine-CMR chamber volumes and systolic function [8–14]. However, to date, fully automated machine learning has not been applied for PC-CMR quantification of valvular flow.

This study tested a novel, machine learning derived, fully automated algorithm for aortic flow quantification on PC-CMR – the goal was to test the incremental utility of machine learning derived (fully automated) flow segmentation to conventional PC-CMR analysis using commercially available automated and manual segmentation. To do so, a broad multicenter cohort of patients undergoing CMR using equipment from different commercial vendors was studied, including patients with coronary artery disease (CAD) as well as an independent cohort enriched for aortic valve pathology.

## Methods

### Model and training

**Fig. 1:**
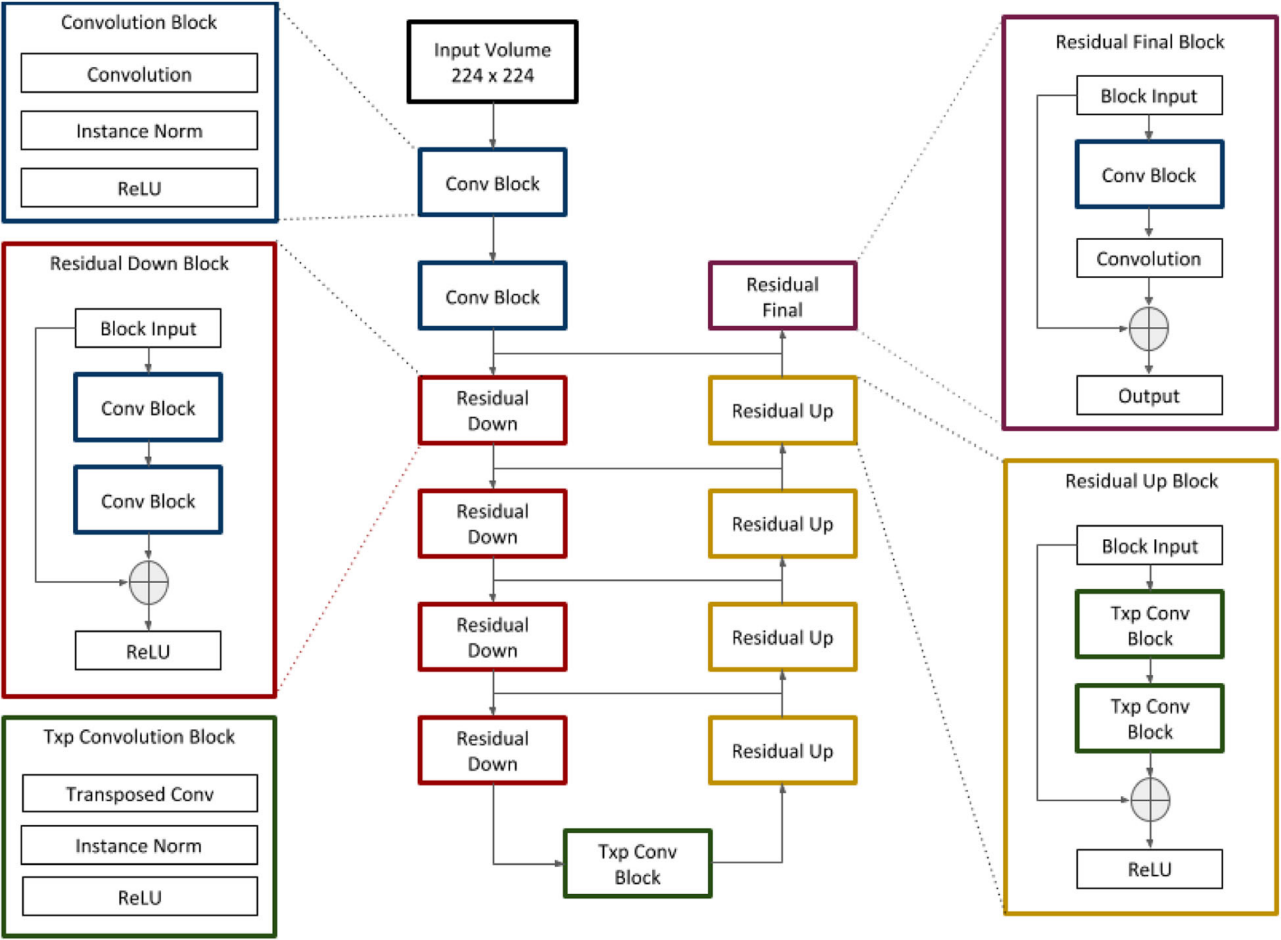
Network Architecture. Schematic illustration of the model, which is based on the U-net architecture. Residual modules improve gradient propagation during training and improve performance

The automated segmentation model was based on neural network architecture described by Han [15], a modified U-net [16], for which excellent performance has been previously demonstrated in medical segmentation: The model makes use of residual modules [17], which improve gradient flow between adjacent layers and increase classification accuracy. A diagram of the model’s architecture is shown in Fig. 1.

**Fig. 2:**
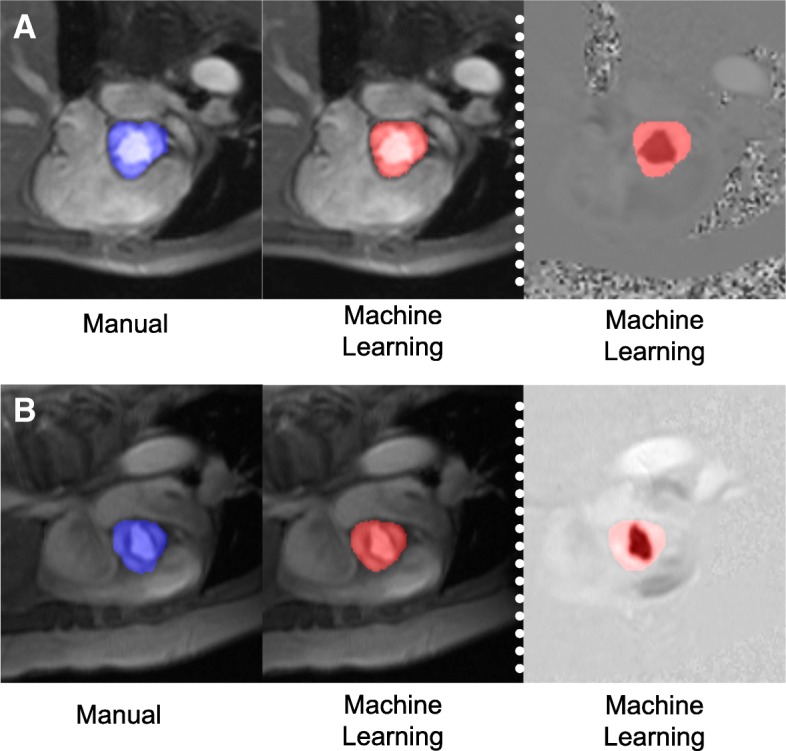
Representative Examples. Typical examples of aortic contouring as performed by manual segmentation (blue, left) and fully automated machine learning (red, right) in a patient with trileaflet (**a**) and bicuspid (**b**) aortic valve. For both examples, magnitude images shown on left, and corresponding PC images shown on right

The machine learning algorithm was initially trained in a *derivation cohort*, consisting of consecutive patients (*n* = 150) who underwent clinical CMR (with aortic valve PC-CMR) between January – November 2017. For each exam, manual segmentation maps were generated from PC-CMR: This entailed labeling pixels in the magnitude images as either valve or non-valve using 3DSlicer [18], an open-source medical image post-processing application. Prior to neural network processing, input images were resampled and (if necessary) zero-padded to 256 × 256 pixels. Pixel intensity values in the magnitude images were then rescaled to values between zero and one. Model training was performed using magnitude images as input and corresponding ground-truth manual segmentation maps as output (Fig. 2). The training set contained a total of 4345 unique images from 150 patients. Aggressive data augmentation was employed at batch time in the form of random zoom, rotation, crop (224 × 224), horizontal/vertical flip, and addition of Gaussian noise. A weighted softmax/cross entropy loss function was used for training as follows:

1 $$ loss\left(x,\kern0.5em i\right)\kern0.5em =\kern0.62em -w\left[i\right]\kern0.5em \ast \kern0.5em \mathit{\ln}\kern0.5em \frac{e^{x\left[i\right]}}{\sum jC{e}^{x\left[j\right]}} $$

where *x* is the output logit vector at a given pixel, *i* the true class label, *w* the vector of class weights, and *C* the number of classes. Weighting was employed to combat class imbalance given that the vast majority of pixels in each image were non-valve. A class weight of 0.2 was empirically assigned to the non-valve class and 0.8 to the valve class. RMSProp was used to apply incremental parameter updates.

The model was built in Python using the deep learning framework Pytorch. Training and testing were performed on a workstation with four CPU cores, 64 GB of system memory, and a graphics processing unit (GPU) with 11 GB of video memory (NVIDIA [Santa Clara, California, USA] GTX 1080 Ti). Software code pertaining to both training and testing of the machine learning model can be found on line at: https://github.com/akbratt/PC_AutoFlow.

### Flow calculation

Data was extracted from PC-CMR DICOM files. Established algorithms were used to convert raw phase map pixel intensities to velocities as follows:

2 $$ v={P}^{\ast }{M}^{\ast } ASF $$

such that

3 $$ ASF=\frac{10\pi R}{VENC} $$

where *P* and *M* represents raw pixel values from the phase and magnitude maps, respectively, *ASF* is an amplitude scaling factor, *R* is a reconstruction scaling factor specified in the DICOM header, and *VENC* is an adjustable scanner parameter representing the maximum measurable flow velocity.

Flow was calculated from the automated segmentation map of a given phase contrast scan as:

4 $$ NetFlow=\kern0.5em \sum \limits_{n=0}^N\sum \limits_{i=0}^I{S}_{n,i}\kern0.5em {V}_{n,i}\kern0.5em a\Delta t $$

where *n* is the segment index, *N* the number of temporal segments in the scan, *I* the number of pixels in each segment, *S* the binary segmentation map, *V* the velocity map calculated using eqs. 1–3, *a* the pixel area (in cm^2^), and *Δt* the time interval between segments.

### Study population

An independent validation cohort was thereafter employed to test the algorithm, which was comprised of CAD patients (*n* = 190) enrolled in two prospective (Cornell) institutional protocols focused on LV remodeling. PC-CMR exams were performed using a standardized protocol, in which PC-CMR datasets were acquired (through plane) at the level of the aortic valve leaflet tips and cine-CMR datasets (for assessment of systolic function) were acquired in contiguous short axis slices (6 mm slice thickness, 4 mm gap) throughout the LV. CMR exams in the validation cohort were performed using a 3T CMR scanner (84% 1.5 T, 16% 3T; General Electric Healthcare, Waukesha, Wisconsin, USA) scanners. Typical PC-CMR parameters were as follows: flip angle = 20 deg., Venc = 150–350 cm/sec, TR [1.5T] = 8 msec, TE [1.5T] = 3.7 msec, TR [3T] = 5 msec, TE = 3.6 msec. Transthoracic echocardiography was performed within one week of CMR (99% within 24h) in accordance with standardized protocol as previously detailed for each of the two prospective studies from which the current cohort was derived [19, 20]. Clinical and demographic information was prospectively acquired at time of study enrollment.

This research protocol was performed with approval of the Weill Cornell Institutional Review Board (IRB), which approved retrospective analysis of pre-existing datasets utilized for model training (derivation cohort). Validation cohort patients provided written informed consent for research participation.

### Volume overlap and surface distance metric analysis

The automated segmentation model was evaluated in terms of volume overlap and surface distance metrics by comparing automated segmentation maps to corresponding ground-truth manual segmentation maps. Volume overlap and surface distance metrics were tested on all scans in the derivation cohort (*n* = 150) using six-fold cross-validation. Cross validation is a procedure whereby data is randomly split into non-overlapping subsets such that a model can be trained on all but one subset and tested on the remaining subset. In this case, a different model instance was trained and tested for each of the 6 hold-out subsets and test metrics were averaged per-case for the entire dataset.

Volume overlap metrics (Dice and Jaccard coefficients) consider only pixels that are labeled as valve. These coefficients take on values between zero and one such that a value of one is perfect overlap between segmentation maps and zero is no overlap. Distance metrics (Hausdorff [HD] and average symmetric surface [ASSD] distances) operate on binary surface plots generated from volumetric segmentations by zeroing any valve pixels with no neighboring non-valve pixels. Equations for volume overlap and surface distance metrics are shown in Additional file 1.

### Flow comparisons / algorithm evaluation

Net forward trans-aortic flow calculated via fully automated machine learning segmentation was compared to that generated by manual segmentation, which was performed by an experienced (level III trained) physician (JWW). Flow differences between manual and machine learning were compared to those between manual and a conventional (commercially available) automated algorithm (Cardiac VX, General Electric Healthcare, Waukesha, Wisconsin, USA): The commercial algorithm requires a user to manually contour a single temporal segment; the segmentation mask is then propagated to all other temporal segments with automatic adjustments to account for valve motion and deformation. To directly test incremental utility of the machine learning approach, analyses using the commercial algorithm were in no way adjusted following initial segmentation.

Intra- and inter-reader reproducibility for manual, conventional automated, and machine learning segmentation were determined via analysis of a random subset of 20 patients.

### External validation

Data from an additional institution (Duke) was used to further test robustness of the model, representing 130 CMR scans acquired using different vendor (Siemens, Munich, Germany]) equipment (53% 3T, 47% 1.5T) in a cohort enriched for patients (*n* = 40) with clinically documented aortic valve pathology (bicuspid aortic valve [BAV]) or aortic stenosis [AS]; 25% mild / 35% moderate / 40% severe). To do so, a new instance of the model was trained on a dataset consisting of the entire derivation cohort (*n* = 150) as well as 50 exams from the external validation cohort (including 10 with AS and 10 with BAV; total *n* = 200). The model was then tested on the remainder of the external validation cohort (*n* = 80), including an equivalent number of patients with aortic valve pathology (*n* = 10 AS, n = 10 BAV). The Duke IRB provided approval for analysis of de-identified datasets for research purposes.

### Statistical methods

Comparisons between groups were made using Student’s t-test (expressed as mean ± standard deviation [SD]) for continuous variables. Inter and intra-observer agreement between methods was assessed using the method of Bland and Altman [21], which yielded mean difference as well as limits of agreement between methods (mean ± 1.96 SD). Bivariate correlation coefficients, intra-class correlation coefficients, and linear regression equations were used to evaluate associations between variables. Statistical calculations were performed using SPSS 24.0 (Statistical Package for the Social Sciences, International Business Machines, Inc., Armonk, New York, USA), SciPy [22], and Excel (Microsoft Inc., Redmond, Washington, USA). Two-sided *p* < 0.05 was considered indicative of statistical significance.

## Results

### Model training, volume overlap, and surface distance metric analysis

Model training was initially performed in the derivation cohort and deemed complete based on training set Dice coefficient plateau. Volume overlap and surface distance metrics during cross validation demonstrated excellent magnitude of agreement between manual and model generated segmentations (mean Dice = 0.940 [CI 0.937–0.943], Jaccard = 0.888 [CI 0.883–0.893], HD = 3.5 mm [CI 3.1–3.9], ASSD = 0.7 mm [CI 0.6–0.8]).

### Clinical application and external validation

Machine learning PC-CMR segmentation was subsequently tested in a cohort of 190 CAD patients undergoing CMR using commercial (GE) scanners, for whom demographics are reported in Table 1. Figure 2 shows representative examples of manual and model-generated data, including cases of tricuspid and bicuspid aortic valve. Segmentation was successful in all cases and required no user intervention.

**Table 1:** Patient characteristics

	Overall (*n* = 190)
Clinical	
Age (years)	57 ± 12
Male gender	87% (165)
Body surface area	2.0 ± 0.2
Coronary Artery Disease Risk Factors	
Hypertension	47% (90)
Hypercholesterolemia	54% (102)
Diabetes mellitus	28% (53)
Tobacco use	35% (66)
Family history	30% (56)
Cardiovascular Medications	
Beta-blocker	91% (173)
ACEI/ARB	60% (113)
Loop diuretic	15% (28)
Statin	93% (177)
Aspirin	98% (186)
Thienopyridine	83% (158)
Warfarin	5% (9)
Nitroglycerin	13% (25)
Cardiac morphology/function	
Left Ventricle	
Ejection fraction (%)	52.2 ± 13.3
LV dysfunction (EF < = 55%)	55% (105)
End-diastolic volume (ml)	161.9 ± 49.2
End-systolic volume (ml)	81.6 ± 46.4
Myocardial mass (g)	137.9 ± 38.2
Late gadolinium enhancement (present)	98% (186)
Infarct size (% myocardium)	14.5 ± 10.4
Aortic Valve	
Bileaflet	2% (3)
Thickening/ fibrocalcific changes	12% (23)
Stenosis	2% (4)
Regurgitation	7% (13)

*ACEI* angiotensin converting enzyme inhibitor, *ARB* angiotensive receptor blocker

**Fig. 3:**
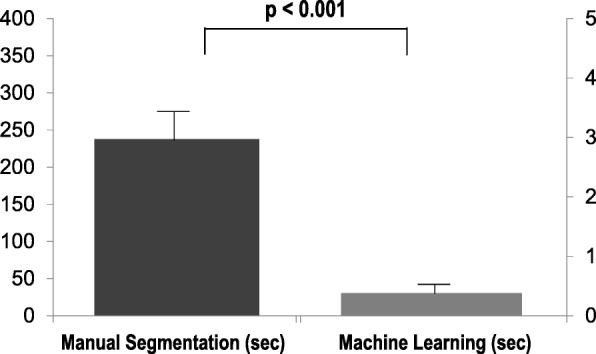
Processing Times. Processing times for manual segmentation and fully-automated machine learning algorithm among validation cohort (data shown as mean ± SD). As shown, mean processing times were > 100-fold lower using machine learning, which processed each case in ~ 380 msec per dataset, corresponding to a total processing time of 1.2 min for the entire validation cohort (*n* = 190)

As shown in Fig. 3, segmentation time was < 0.01 min per case for machine learning segmentation (1.2 min for entire dataset) with GPU acceleration, whereas manual segmentation required an average time of 3.96 ± 0.36 min per case (12.5 h for entire dataset). Automated per-case segmentation time averaged 19.04 s without GPU acceleration.

### Reproducibility

Table 2 reports intra- and inter-observer variability (mean difference in net flow) for manual, conventional automated, and machine learning segmentation methods. As shown, both manual and conventional automated methods yielded good reproducibility, as evidenced by non-significant mean differences and small limits of agreement as well as high intra-class correlation coefficients (ICC) for manual (inter- and intra-rater ICC both > 0.99) and conventional automated (both > 0.97) segmentations. Since the machine learning model is deterministic with respect to input (apart from correctly loading DICOMs for a given dataset), inter- and intra-observer variability for model-generated segmentations was zero and intraclass correlation coefficients were 1.0.

**Table 2:** Reproducibility Analyses

	Intra-Observer			Inter-Observer		
	Mean ± SD (mL)	Limits of Agreement (mL)	*p*	Mean ± SD (mL)	Limits of Agreement (mL)	*p*
Manual	0.18 ± 1.6	−3.0 to 2.5	0.32	−0.28 ± 1.2	−2.6 to 2.1	0.62
Conventional	−0.33 ± 1.8	−3.8 ± 3.1	0.42	0.28 ± 3.0	- 5.7 ± 6.3	0.69
Machine Learning	0	0	–	0	0	–

### Comparisons to conventional flow segmentation

**Fig. 4:**
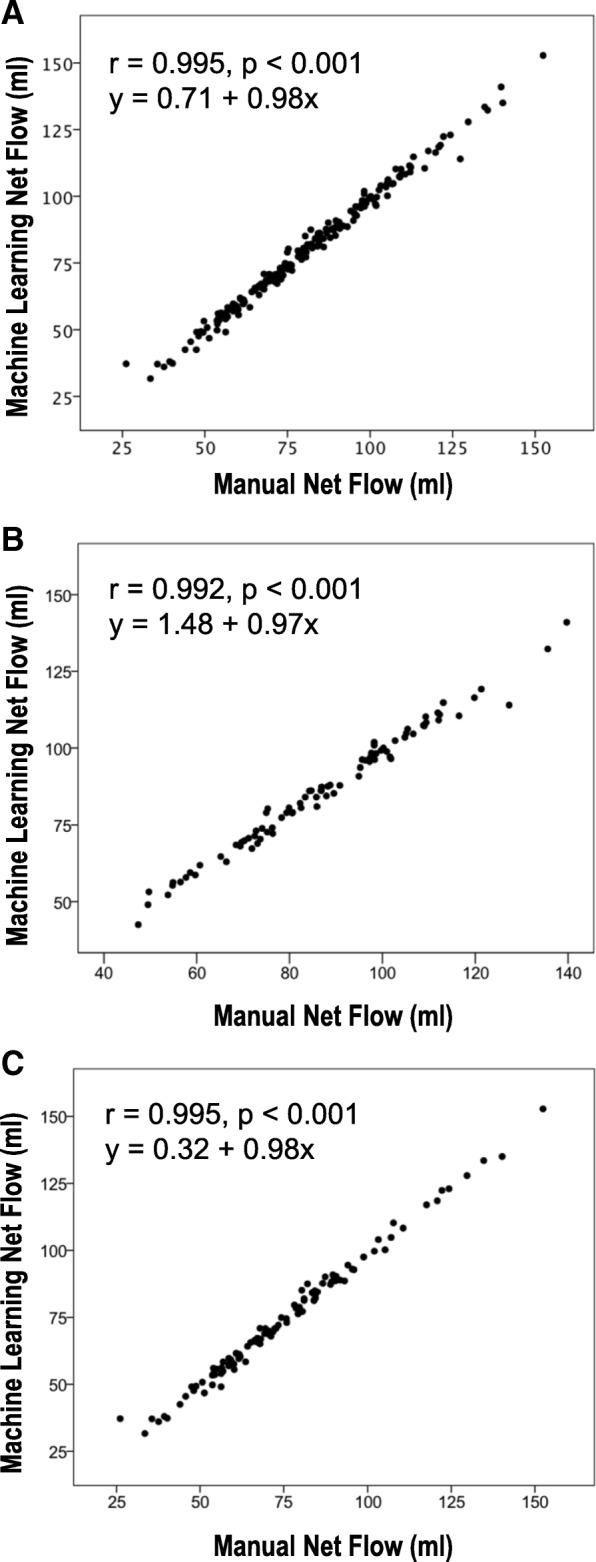
Correlations Between Machine Learning and Manually Processed Flow. Scatter plots demonstrating correlations between fully-automated machine learning and manually processed flow among the overall study cohort (**a**) as well as among subgroups of patients with (**b**) and without (**c**) preserved left ventricular ejection fraction (LVEF≥55%). Note correlations approaching near unity (*r* > 0.99) in all groups

As illustrated in Fig. 4, correlations between machine learning- and manual segmentation-derived flow approached unity (*r* = 0.99) among the overall study cohort, as well as among patients with (*n* = 102) and without (*n* = 88) LV systolic dysfunction (LV ejection fraction [EF] < 55%). Table 3 details flow quantification results by respective segmentation methods. As shown, both machine learning and conventional automated methods yielded extremely small, albeit statistically significant absolute differences vs. manual segmentation, although magnitude of difference was smaller for machine learning vs. conventional automation (1.85 ± 1.80 vs. 3.33 ± 3.18 mL, *p* < 0.01). Machine learning also yielded a slightly higher intra-class correlation coefficient in relation to manual segmentation (ICC = 0.994) than did conventional automated segmentation (ICC = 0.980).

**Table 3:** Difference in net flow between manual segmentation in relation to machine learning and conventional (commercially available) automated segmentation

	Net Flow	Absolute Difference (|manual – method|)	Correlation
Manual	81.5 ± 24.2 mL		
Machine Learning	80.5 ± 23.7 mL^a^	1.85 ± 1.80 mL^b^	y = 1.01x + 0.16 r^2^ = 0.99, *p* < 0.001
Conventional	80.1 ± 23.2 mL^a^	3.33 ± 3.18 mL^b^	y = 1.02x – 0.31 r^2^ = 0.96, *p* < 0.001

^a^Both *p* < 0.01 (segmentation method vs. manual)

^b^p < 0.01 (machine learning vs. conventional segmentation in terms of MAD)

**Fig. 5:**
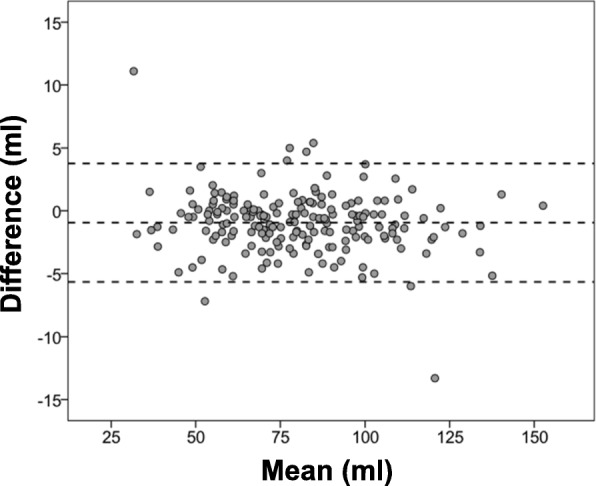
Bland-Altman Plot. Bland-Altman plots comparing fully automated machine learning to manually processed flow tracing for the overall study cohort. Middle line denotes mean. Dashed lines denote ±1.96 standard deviations

Regarding methodological differences, Fig. 5 reports Bland-Altman plots for machine learning data in relation to manual segmentation: As shown, 98% (*n* = 186) of cases differences were ≤ 5 mL: In 2 cases with marked discrepancies (~ 10 mL), prominent artifact (1 prominent peri-aortic dephasing artifact, 1 motion artifact) may have been responsible. Notably, in the two cases for which machine learning yielded greatest difference with manual segmentation, conventional automated and manual methods also yielded a substantial difference in one case (12.5 mL) and a lesser difference in the other (3.6 mL).

### Performance in relation to LV dysfunction and mitral regurgitation

**Fig. 6:**
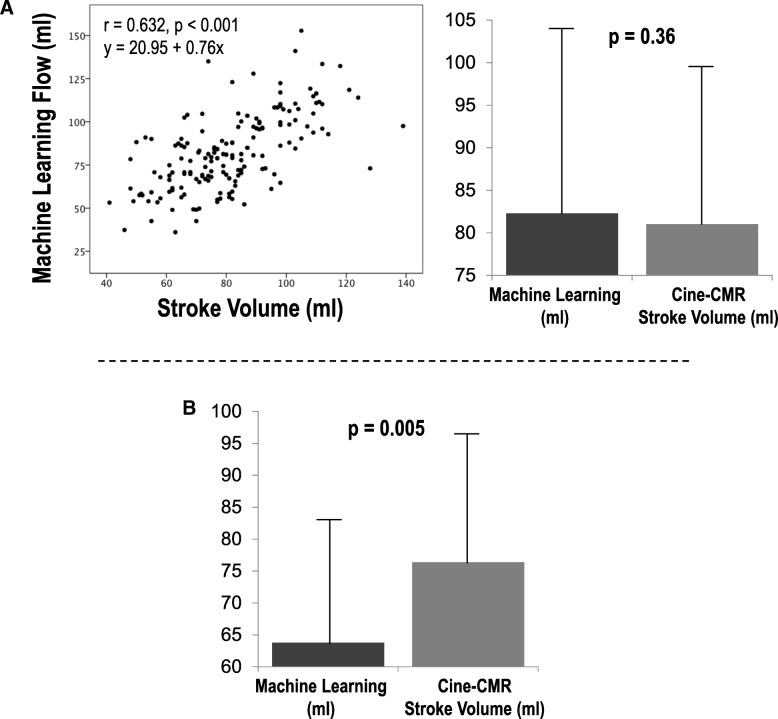
Machine Learning Derived Aortic Flow in Relation to Cine-CMR LV Stroke Volume. **a** Correlations between aortic through-plane flow as quantified by fully automated machine learning algorithm and LV volumetric stroke volume as quantified on cine-CMR among patients without advanced (>mild) mitral regurgitation. Note moderate correlation between approaches (left) and non-significant differences in stroke volume (right). **b** Machine learning aortic flow and cine-CMR stroke volume among patients with advanced (>mild) mitral regurgitation. Note lower transaortic flow as quantified by cine-CMR consistent with decreased forward systemic output in context of mitral regurgitation.

Cine-CMR stroke volume was employed as an independent means of assessing machine learning derived aortic flow; echocardiography was used as an independent arbiter for presence/severity of mitral regurgitation. As shown in Fig. 6a, correlations between the two approaches were good (*r* = 0.63, *p* < 0.001) among patients without advanced (>mild) mitral regurgitation (a known cause for differential volumetric and forward LV stroke volume) – paralleling small, non-significant differences in mean stroke volume (∆ 1.3 ± 17.7 mL, *p* = 0.36). Manual and conventional automated methods yielded similar magnitude of correlation with LV volumetric stroke volume (*r* = 0.65, *p* < 0.001 for both), paralleling non-significant differences in LV stroke volume as calculated by cine-CMR volumetric segmentation and respective PC-CMR segmentation methods (both p = NS).

As shown in Fig. 6b, machine learning-derived segmentations yielded lower stroke volume than did volumetric cine-CMR data among patients with advanced (>mild) mitral regurgitation (∆ 12.6 ± 20.9 mL, *p* = 0.005), consistent with decreased forward cardiac flow in context of atrio-ventricular valvular insufficiency.

### External validation

Further evaluation of the machine learning algorithm in the external (Siemens) dataset demonstrated equivalent model performance. There was excellent agreement between manual and automated segmentation in the external validation cohort with respect to volume overlap (mean Dice = 0.940 [CI 0.936–0.944], mean Jaccard = 0.887 [0.881–0.893]). Similarly, manual and machine learning derived flow measurements yielded small mean difference in net flow (∆ 1.39 ± 1.77 mL, *p* = 0.8) and high magnitude of correlation (*r* > 0.99, *p* < 0.001). Among the subgroup of 20 patients with aortic valve pathology encompassed in the test set of the validation cohort (*n* = 10 BAV, n = 10 AS), machine learning yielded similarly small mean differences (∆ 1.71 ± 2.25 mL, *p* = 0.25) and high correlations (r > 0.99, p < 0.001) with manual segmentation.

## Discussion

This study—testing a novel application of machine learning for fully automated aortic flow quantification—demonstrates several key findings: First, machine learning performed robustly among a broad cohort of CAD patients, as evidenced by successful segmentation in all cases and rapid processing time (*n* = 190 cases, cumulative time 1.2 min [600-fold shorter than manual segmentation]), as well as close correlations (*r* = 0.99) and small mean differences (1.85 ± 1.80 mL) in flow as quantified manually by an expert CMR reader. Second, machine learning outperformed a conventional automated segmentation algorithm, yielding lower error and error variance with respect to manual segmentation (1.85 ± 1.80 vs 3.33 ± 3.18 mL). Third, machine learning was robust with respect to data source; model performance was excellent when applied to two independent institutional datasets (inclusive of CAD and aortic valve pathology patients) acquired on different CMR scanners. Fourth, machine learning aortic PC-CMR segmentation yielded good correlation (*r* = 0.63, p < 0.001) and non-significant mean differences with cine-CMR derived LV stroke volume in patients without advanced MR: Among patients with advanced mitral regurgitation, machine learning-derived flow was lower than was volumetric LV stroke volume (∆ 12.6 ± 20.9 mL, *p* = 0.005), further supporting physiologic validity of aortic flow as quantified by our automated segmentation algorithm.

It is important to note that the machine learning approach developed in our study requires no human supervision apart from quality control. In this regard, these results build upon prior work by our group, which has developed fully automated segmentation algorithms for cine-CMR that have been shown to yield superior performance to manual and conventional automated segmentation with respect to clinical robustness, as well as agreement with ex-vivo volumetric phantoms and necropsy-evidenced myocardial mass [23, 24]. In this context, we believe our findings are of substantial relevance to CMR application in large scale-population based datasets.

To the best of our knowledge, this is the first study to apply machine learning for flow quantification on PC-CMR. Prior work has applied deep learning segmentation to the brain, [25, 26] prostate, [27, 28] breast, [29] pulmonary, [7] and musculoskeletal systems [30]. Prior applications of deep learning to CMR have focused on cardiac function/remodeling [8–14], for which analysis has entailed segmentation of cine-CMR (SSFP) datasets. Novelty of our study stems from several different factors: First, our machine learning derived algorithm is focused on PC-CMR - a pulse sequence that is widely applied in clinical practice for which no published CNN-based computer vision model has previously been applied. Second, our model was extensively validated in a broad clinical cohort (inclusive of patients with aortic pathology) imaged at two independent medical centers using CMR scanner equipment from two different vendors – providing confidence that our study findings are robust and broadly applicable. Our model architecture is informed by prior approaches to deep learning analysis of CMR, with specific design and hyperparameter choices tailored to our particular task and dataset. The model makes use of a two-dimensional U-net architecture [16], which applies skip connections between the contracting and expanding pathways of the network to recover fine-grained imaging features during decoding. Residual connections [17] were added to improve the performance of the network by use of identity mappings, which prevent vanishing and exploding gradients during training. Network size was chosen to strike a balance between expressivity and overfitting given the modest size of our dataset.

Major advantages of the proposed segmentation model are speed and reproducibility. PC-CMR interpretation is time consuming because it requires painstaking manual segmentation. Even with the aid of existing conventional automated methods, substantial manual corrections are often necessary, negating much of the gained efficiency. Further, manual segmentation is prone to subjective decision-making, which adds to quantitative variability and reduces diagnostic confidence. These drawbacks limit feasibility of large-scale prospective CMR studies, as well as batch analysis of de-identified web-based multicenter registry data (currently > 50,000 exams) currently being accrued by several organizations [31]. The machine learning algorithm tested in this study is well-suited for such applications, given that it is highly reproducible, with speed and accuracy sufficient to process large volumes of data with minimal supervision. Another advantage is the versatility of neural networks with respect to training data. The model described here makes no physical or anatomic assumptions; it simply reproduces the patterns observed in the training set. As a result, with a sufficient volume of data, the model could easily be repurposed to evaluate other populations, valves, and pathologies.

Several limitations should be noted. First is the lack of a gold-standard test with which to evaluate model performance. All metrics were compared with manual segmentation, which is a suboptimal standard given that it is subjective and stochastic. On the other hand, it should be noted that beyond excellent agreement with manual PC-CMR analysis, machine learning derived data was consistent with two independent imaging approaches; volumetric stroke volume as measured on cine-CMR, as well as differential LV volumetric and aortic forward flow among patients with echocardiography evidenced mitral regurgitation.

It is also important to recognize that in current clinical practice, commercial PC-CMR automated segmentation results are reviewed and frequently manually corrected prior to flow calculation. PC-CMR segmentation tools intended for fully automated segmentation are not developed, and thus could not be used as a comparator in our study. We acknowledge that differences between commercial and manual segmentation would likely have been smaller if commercial segmentation analyses were manually corrected on a frame by frame basis. However, this approach would have prohibited direct comparison between standard commercial segmentation technology and our machine learning derived algorithm, which was a goal of our study. More broadly, manual correction following initial automated segmentation is impractical for analysis of large scale CMR datasets providing a strong rationale for development of more accurate, fully automated, solutions as can be fostered by machine learning. Finally, our training dataset was relatively small in size (*n* = 150 [derivation] and *n* = 200 [derivation+external validation]) and included a select group of patients with CAD and/or aortic valve disease. This is due to the fact that manual segmentation is time- and resource-intensive, which makes it difficult to generate data at the scale one expects in large commercial and industrial settings. Ongoing analysis (informed by current findings) is focused on diagnostic and prognostic applications of this machine learning PC-CMR algorithm in broader cohorts.

## Conclusions

The current study provides proof of concept concerning utility of a fully automated deep learning method for PC-CMR aortic flow quantification, demonstrating it to be fast, robust, and superior to that of existing commercial software. The deep learning method described in this manuscript increases interpretive efficiency and reproducibility while maintaining high accuracy, enabling large scale population studies to be performed with minimal supervision. Future work will focus on expanding machine learning to other cardiac valves, and will compare machine learning and manual segmentation to predict clinical prognosis and therapeutic response in patients undergoing CMR.

## Additional file

Additional file 1: Volume Overlap and Surface Distance Equations. (DOCX 14 kb)

## Abbreviations

AS: Aortic stenosis
ASSD: Average symmetric surface distance
BAV: Bicuspid aortic valve
CAD: Coronary artery disease
CMR: Cardiovascular magnetic resonance
CNN: Convolutional Neural Networks
HD: Hausdorff Distance
ICC: Interclass correlation coefficient
LV: Left ventricle/left ventricular
LVEF: Left ventricular ejection fraction
MAE: Mean Absolute Error
PC: Phase contrast
VENC: Velocity Encoding Factor
