# Supplementary material for: Machine learning derived segmentation of phase velocity encoded cardiovascular magnetic resonance for fully automated aortic flow quantification
Source: J Cardiovasc Magn Reson. 2019 Jan 7;21:1. doi: 10.1186/s12968-018-0509-0 (PMC6322266; doi:10.1186/s12968-018-0509-0)
Supplement: Supplementary file 1 — Volume Overlap and Surface Distance Equations. (DOCX 14 kb) [file 12968_2018_509_MOESM1_ESM.docx]

**Additional Files 1**

**Volume Overlap and Surface Distance Equations**

*Dice Coefficient*:

The Dice coefficient is calculated as:

| $DICE\left( A,B \right)=\frac{2\left\vert A\cap B \right\vert}{\left\vert A \right\vert+\left\vert B \right\vert}$ |  |
| --- | --- |

where A and B are binary segmentation maps.

*Jaccard Coefficient*:

The Jaccard coefficient (or intersection over union [IOU]) is given by:

| $IOU\left( A,B \right)=\frac{\left\vert A\cap B \right\vert}{\left\vert A\cup B \right\vert}$ |  |
| --- | --- |

*Symmetric Hausdorff Distance (HD)*:

Intuitively, the symmetric Hausdorff distance, or maximum surface distance, is the maximum euclidean distance from a point in one surface map to the nearest point in another surface map. Concretely:

| $HD\left( A_{s},B_{s} \right)=max\text{\{}\sup_{a\in A}\inf_{b\in B}d\left( a,b \right),\sup_{b\in B}\inf_{a\in A}d\left( a,b \right)\text{\}}$ |  |
| --- | --- |

where A_s_ and B_s_ are surface plots for binary segmentation maps A and B, sup is the supremum, inf the infimum, and d(a, b) the euclidean distance function.

*Average Symmetric Surface Distance (ASSD)*:

The average symmetric surface distance is similar to the SHD except that it uses the mean instead of the maximum/supremum:

| $ASSD\left( A_{s},B_{s} \right)=\frac{1}{\left\vert A_{s} \right\vert+\left\vert B_{s} \right\vert}\left( \sum_{a\in A_{s}} \inf_{b\in B_{s}}d\left( a,b \right)+\sum_{b\in B_{s}} \inf_{a\in A_{s}}d\left( a,b \right) \right)$ |  |
| --- | --- |
